# *Enterococcus faecium* HDRsEf1 Promotes Systemic Th1 Responses and Enhances Resistance to *Salmonella* *Typhimurium* Infection

**DOI:** 10.3390/nu15194241

**Published:** 2023-09-30

**Authors:** Jin Zhou, Tingyang Wang, Lele Fan, Hongde Xiao, Hui Ji, Naiji Zhou, Zutao Zhou, Huazhen Liu, Muhammad Akhtar, Yuncai Xiao, Deshi Shi

**Affiliations:** 1 State Key Laboratory of Agriculture Microbiology, College of Veterinary Medicine, Huazhong Agricultural University, Wuhan 430070, China; jinzhou@webmail.hzau.edu.cn (J.Z.); wangtingyang@webmail.hzau.edu.cn (T.W.); fanlele0525@163.com (L.F.); xhd0816@163.com (H.X.); hui_ji0325@163.com (H.J.); zhounaiji@126.com (N.Z.); ztzhou@mail.hzau.edu.cn (Z.Z.); 2 Key Laboratory of Development of Veterinary Diagnostic Products, Ministry of Agriculture, Huazhong Agricultural University, Wuhan 430070, China; 3 Key Laboratory of Agricultural Animal Genetics, Breeding and Reproduction of Ministry of Education, Huazhong Agricultural University, Wuhan 430070, China; hz219@mail.hzau.edu.cn (H.L.); akhtarshakir122@gmail.com (M.A.)

**Keywords:** gut microorganisms, *Enterococcus faecium*, spleen, immunomodulation, Th1 cells, *Salmonella Typhimurium*

## Abstract

The gut microbiota is known to regulate the immune system and thereby influence susceptibility to infection. In this study, we observed that the administration of *Enterococcus faecium* HDRsEf1 (HDRsEf1) led to an improvement in the development of the immune system. This was evidenced by an increase in both the spleen index and the area of spleen white pulp. Specifically, the proportion of T helper (Th) 1 cells and the production of IFN-γ and IL-12 were significantly increased in the spleens of mice treated with HDRsEf1. In agreement with the in vivo results, we found that Th1-related cytokines, including IFN-γ and IL-12p70, were strongly induced in splenocytes treated with HDRsEf1. In addition, Th1 cell activation and high-level secretion of IL-12p70 were also confirmed by coculture of CD4^+^ T cells with bone marrow-derived dendritic cells treated with HDRsEf1. Moreover, the employment of HDRsEf1 was identified to augment resilience against systemic infection provoked by *S*. *Typhimurium* and stimulate the expression of the genes for TNFα and iNOS in the initial stage of infection, signifying that reinforced Th1 cells and IL-12 might activate macrophages for antibacterial safeguards. In summary, our study suggests that HDRsEf1 could act as an effective immunobiotic functional agent, promoting systemic Th1 immunological responses and priming defenses against infection.

## 1. Introduction

The intestinal tract of animals contains a diverse community of microorganisms, some of which are crucial in developing and regulating the immune system [1]. Research findings have indicated that the composition of the gut microbiota has a significant correlation with the various subtypes of CD4^+^ T cells, consequently influencing vulnerability toward different types of pathogens [2,3,4]. Th1 cells and Th17 cells are regulated by the gut microbiota and thereby influence antibacterial defenses against intracellular and extracellular bacterial infections, respectively [3,5]. Furthermore, symbiotic microorganisms have the potential to impact host immune cells beyond the intestinal mucosal interface. Microbe-associated molecular patterns (MAMPs) and small molecular metabolites from the intestine can enter the circulatory system and interact with immune cells. This interaction extends their influence to extraintestinal organs, including the liver, spleen, and thymus [6,7,8,9,10]. Furthermore, the gut microbiota also partakes in the process of instructing immune cells within the intestinal mucosa, subsequently relocating to various organs outside the gut to aid in bolstering overall host defense [11]. Probiotics are live microorganisms that can provide health benefits to the host when consumed in sufficient amounts [12]. They work by competing with harmful microorganisms to prevent adhesion in the intestinal epithelium or by activating the immune system to enhance resistance to pathogenic microorganisms [13,14,15,16]. Therefore, the development of immunomodulatory probiotics presents an opportunity to regulate the immune system for long-lasting health benefits. *Enterococcus faecium* is a symbiotic microorganism that resides in the intestines of humans and other mammals, and it has been employed as a probiotic in medicine and animal husbandry to tackle bacterial infections for several decades [17]. Previous studies have attributed the probiotic effects of *Enterococcus faecium* to its ability to directly inhibit pathogenic bacteria, enhance intestinal barrier function, and suppress intestinal inflammation [18,19,20]. However, how *Enterococcus faecium* regulates systemic immune function and improves host defenses against invasive systemic infections has not been fully studied.

*Enterococcus faecium* HDRsEf1 (HDRsEf1) is a Chinese national patent strain isolated from the intestinal contents of Chinese local breed Tongcheng pigs. It has been found to exhibit probiotic characteristics in animals, and in vitro studies have suggested that HDRsEf1 could alleviate intestinal inflammation and promote the expression of tight junction proteins [21,22,23,24,25]. The objective of this research is to examine the influence of HDRsEf1 on the regulation of systemic immunity, specifically with regards to CD4^+^ T cells. Our findings indicate that HDRsEf1 promotes spleen development and stimulates the production of IL-12 and T helper (Th) 1 cells in the spleen. Th1 cells and IL-12 have been shown to program macrophages for potential antibacterial defenses [26,27,28]. Therefore, we further investigated the potential of HDRsEf1 in protecting the host against systemic infection caused by *Salmonella enterica* serotype Typhimurium (*S. Typhimurium*). *S. Typhimurium* is an intracellular pathogen that spreads systemically and survives within macrophages located in systemic sites [29]. In this study, we found that HDRsEf1 promoted early protection against systemic infection caused by *S. Typhimurium* and enhanced the expression of bactericidal genes in the spleen during the early stages of infection.

## 2. Materials and Methods

### 2.1. Bacterial Strain and Growth Conditions

HDRsEf1 was isolated from the intestinal contents of Tongcheng pigs, a Chinese local breed. *Enterococcus faecium* NY5 and *Lactobacillus salivary* CF10, used as controls, were isolated from the intestinal contents of pigs and chickens, respectively. These strains were cultured in Mann-Rogosa Sharpe Agar (MRS, Hopebio, Qingdao, China) at 37 °C for 24 h. For the purpose of systemic infection, *S*. *Typhimurium* (ST, ATCC 14028) was selected and cultured in *Salmonella–Shigella* agar (SS, Hopebio, Qingdao, China) at 37 °C under aerobic conditions.

### 2.2. Animals

The Experimental Animal Center of Huazhong Agricultural University provided female C57BL/6N mice at an age range of 4–5 weeks. The mice were kept in a specific pathogen-free environment with a 12-h light–dark cycle throughout the experiment. They were given unrestricted access to both food and water. The animal care and use protocols adhered to the Guidelines for the Care and Use of Animals for Research and Teaching and received approval from the Animal Care and Use Committee of Huazhong Agricultural University (permit number: HZAUMO-2023-0123).

### 2.3. Feeding Probiotic

After adaptation for 1 week, the mice were randomly divided into 2 groups (*n* = 15 for each group): a control group and an HDRsEf1 group. HDRsEf1 in an exponential phase was harvested via centrifugation at 3000× *g* and rinsed two times using phosphate-buffered saline (PBS). A suspension of 1 × 10^8^ CFU HDRsEf1 in 100 µL PBS was administered to the mice via intragastric gavage for 10 consecutive days, while an equal volume of sterile PBS was given to the control group. The presence of *Enterococcus* in mice feces was detected using *Enterococcus* agar (Hopebio, Qingdao, China) every 2 days. At the end of the experiment, the spleens of the mice were collected for weighing and morphological analysis, and spleen cells were separated for flow cytometry analysis.

### 2.4. Histological Tissue Analysis

Paraformaldehyde was utilized to fix the tissue, followed by embedding it in paraffin. The ensuing sections of 4 µm thickness were subjected to staining with hematoxylin and eosin (H&E). To capture the images, a panoramic MIDI slide scanner (3D HISTECH Limited, Budapest, Hungary) was employed. The measurement of the intact white pulp area was conducted in five representative regions of each specimen using Image-Pro Plus 6.0 (Media Controls, Inc., Bethesda, MD, USA).

### 2.5. Flow Cytometry Analysis

The spleen was extracted from each mouse and processed to obtain a single-cell suspension through grinding and filtering. The suspension underwent treatment with red blood cell lysate for 1 min to eliminate red blood cells. Subsequently, the cells were stained with Fixable Viability Stain 780 to identify viable cells, as well as anti-mouse monoclonal antibodies, including CD45-APC-Cy7, CD3-FITC, CD4-BV421, and CD8-PerCP-Cy5.5 (all obtained from BD Pharmingen, Franklin Lakes, NJ, USA), to label the surfaces of the cells. To analyze the different subsets of T helper (Th) cells, spleen cells were exposed to phorbol 12-myristic acid-13 acetate (PMA) and ionomycin in the presence of brefeldin A (Leukocyte Activation Cocktail; BD Pharmingen, Franklin Lakes, NJ, USA) for a duration of 6 h. Following this stimulation, the cells were stained with anti-mouse CD4-BV421, IFN-γ-PE-Cy7, IL-4-APC, and IL-17-PE monoclonal antibodies (also obtained from BD Pharmingen, Franklin Lakes, NJ, USA). Fluorescence expression was analyzed using a flow cytometric system (Beckman Coulter, Miami, FL, USA), and the flow cytometric data were acquired and processed using FlowJo software (version 10.8.1. Tree Star, Ashland, OR, USA). To detect the presence of cytokines in the supernatant, splenocytes were cultured in RPMI-1640 complete medium within a 24-well culture plate (2 × 10^6^ cells/well) for a duration of 3 days, with the addition of soluble anti-CD3 monoclonal antibody (1 μg/mL, BioXcell, West Lebanon, NH, USA). The concentrations of IL-4, IL-6, IL-10, IL-12, TNFα, MCP-1, IL-17, and IFN-γ in the supernatant were quantified using a Cytometric Bead Array (BD Pharmingen, Franklin Lakes, NJ, USA) and analyzed using FCAP software 3.0.1 (BD Biosciences, Franklin Lakes, NJ, USA).

### 2.6. In Vitro Splenocyte Stimulation

In a 24-well culture plate, splenocytes derived from C57BL/6N mice were cultivated using complete RPMI-1640 medium (2 × 10^6^ cells/well). The splenocytes were then stimulated with heat-inactivated HDRsEf1 at various cell to bacteria ratios (10:1, 1:1, 1:10), LPS (100 ng/mL; Sigma-Aldrich, Saint Louis, MO, USA), or soluble anti-CD3 monoclonal antibody (mAb) (1 µg/mL, BioXcell, West Lebanon, NH, USA) for 3 days. After 3 days, the culture supernatant was collected for cytokine analysis.

### 2.7. Coculture of CD4^+^ T and Bone Marrow-Derived Dendritic Cells (BMDCs)

In order to obtain bone marrow cells, we isolated femora and tibiae from 6-week-old female C57BL/6N mice. Then, the bone marrow cells were separated and cultured as previously described [30]. After undergoing filtration and treatment to lyse red blood cells, the bone marrow cells underwent PBS rinsing and suspension in RPMI-1640 solution. This medium contained 20 ng/mL of granulocyte macrophage colony-stimulating factor (GM-CSF; ABclonal, Wuhan, China) and 10 ng/mL of IL-4 (ABclonal, Wuhan, China). The cells were then added to a 24-well culture plate at a concentration of 2 × 10^6^ cells per well. Incubation of the cells at 37 °C was carried out for a period of 7 days. Every 2 days, half of the volume of RPMI-1640 medium containing cytokines was replaced. The isolation of naïve CD4^+^ T cells was conducted by following the Naïve CD4^+^ T Cell Isolation kit (Miltenyi Biotec, San Diego, CA, USA) in C57BL/6 mice spleens. Following the isolation process, a coculture of these cells with BMDCs was performed at a ratio of 5:1. The CD4^+^ T cells and BMDCs were then stimulated with either heat-inactivated HDRsEf1 (at a ratio of 1:3 cells to bacteria), LPS (100 ng/mL; Sigma-Aldrich, Saint Louis, MO, USA), or soluble anti-CD3 monoclonal antibody (at a concentration of 1 µg/mL) for a duration of 3 days. Finally, the supernatant was collected and analyzed for major cytokine reactions.

### 2.8. Quantitative Expression Analysis by RT-PCR

In this study, we employed Trizol reagent (Aidlab Biotech, Beijing, China) for the extraction of RNA from intestinal and spleen tissues. The quantification of RNA was carried out for cDNA synthesis. For reverse transcription, 1 µg of total RNA was subjected to the RT Kit with gDNA Clean (Accurate Biotechnology (Hunan) Co., Ltd., Changsha, China), following the manufacturer′s guidelines. To perform quantitative real-time PCR, a reaction volume of 20 µL was utilized, comprising 2 µL of cDNA, 10 µL of 2× SYBR Green Master Mix (Accurate Biotechnology (Hunan) Co., Ltd., Changsha, China), 7 µL of double-distilled H_2_O, and 0.5 µL of each primer. The primers used for the assessment of inflammatory cytokines (IL-6, IL-12, TNF-α), iNOS, and β-actin can be found in Table S1. In this analysis, the mRNA levels of the studied genes were normalized to β-actin mRNA levels, and the subsequent data were analyzed employing the 2^−∆∆CT^ method.

### 2.9. Challenging with S. Typhimurium

After adaptation for 1 week, the mice were randomly divided into 2 groups (*n* = 15 for each group): an ST group and an HDRsEf1 + ST group. The mice were treated with PBS or HDRsEf1 every day for 10 days before bacterial infection. For the *S*. *Typhimurium* infection test, *S*. *Typhimurium* in an exponential phase was collected, washed, and suspended in PBS. Then, all mice were infected with *S*. *Typhimurium* through oral gavage with 1 × 10^7^ CFU per mouse. Similarly, 10 mice were randomly divided into 2 groups (*n* = 5 for each group); the mice were treated with PBS or HDRsEf1 every day for 10 days and then infected with *S*. *Typhimurium* through intravenous injection with 1 × 10^2^ CFU per mouse. All animals were euthanized 96 h after the challenge.

### 2.10. Investigation of Bacterial Colonization in the Liver and Spleen

After euthanasia, each mouse’s liver and spleen were carefully removed and weighed. Under strict aseptic conditions, a homogenizer was utilized to homogenize the collected samples. The resulting homogenate was subsequently diluted using PBS. The diluted solution was then subjected to inoculation onto SS agar plates and incubated at a temperature of 37 °C for a duration of 24 h. Finally, the distinctive colonies were enumerated to assess bacterial colonization.

### 2.11. Statistical Analysis

The data analysis was performed utilizing SPSS software (version 20, Chicago, IL, USA). The findings are displayed in terms of mean ± SEM or median. To compare two groups, Student’s *t*-test or the Mann–Whitney test were employed, while for multiple comparisons, one-way analysis of variance (ANOVA) followed by the Bonferroni test was utilized. Statistical significance was defined as *p* < 0.05, *p* < 0.01, and *p* < 0.001.

## 3. Results

### 3.1. HDRsEf1 Promotes Spleen Development

**Figure 1:**
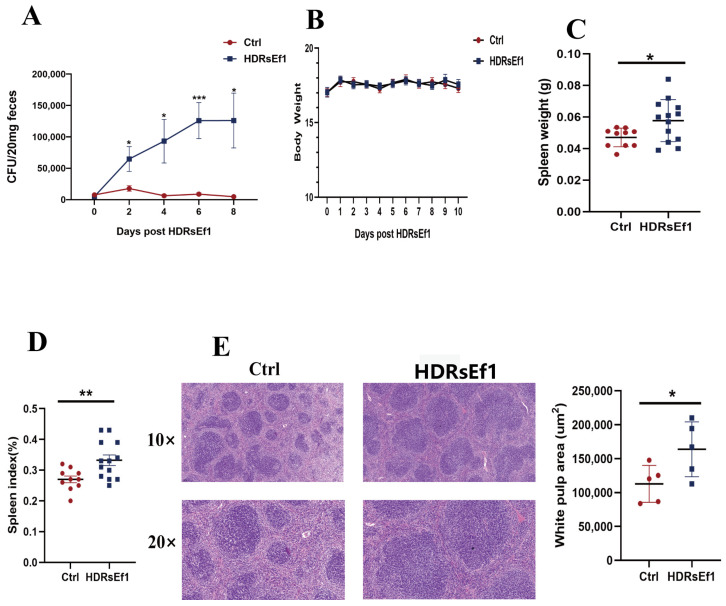
HDRsEf1 promotes spleen development. HDRsEf1 or PBS were orally administered once daily for 10 days. (**A**) The number of *Enterococcus* in the feces from the mice in different groups (*n* = 12–15). (**B**) The effects of HDRsEf1 on the body weight of the mice (*n* = 15). (**C**,**D**) Spleen weight and spleen index in both groups (*n* = 10–13). (**E**) Histomorphological analysis of the spleen using H&E staining (*n* = 5). Data are representative as mean ± SEM. Student’s *t*-test, * *p* < 0.05, ** *p* < 0.01, *** *p* < 0.001.

In this investigation, we first evaluated the capacity of HDRsEf1 to colonize the intestinal tract of mice. Using *Enterococcus* selective agar, we quantified the colony-forming units (CFU) of *Enterococcus* in mouse feces. Our results showed that the CFU of *Enterococcus* in feces gradually increased with the oral administration of HDRsEf1 and was significantly higher than that in the control group by more than 10 times on the eighth day (Figure 1A). This indicates that HDRsEf1 can successfully colonize in the intestinal tract of mice, despite the presence of competitive normal flora. After colonizing HDRsEf1 for 10 days, there was no significant change in the body weight of the mice. However, there was a noticeable increase in spleen weight and spleen index, with the spleens being 20% heavier than those of the control group (Figure 1B–D). Next, histomorphological changes of the spleen in mice treated with HDRsEf1 were evaluated using H&E staining. The results showed that the spleens of mice treated with HDRsEf1 had a significantly larger area of white pulp, which was composed of lymphocytes (Figure 1E). Therefore, it can be concluded that HDRsEf1 effectively colonizes the intestinal tract and promotes immune development.

### 3.2. HDRsEf1 Promotes Th1 Differentiation and Function in the Spleen

**Figure 2:**
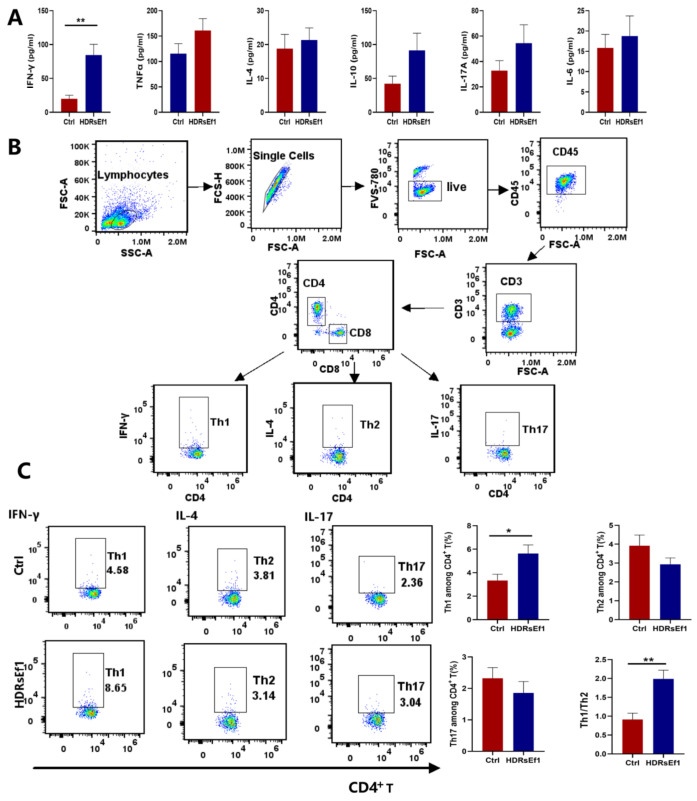
HDRsEf1 promotes T helper (Th) 1 differentiation and function in the spleen. (**A**) The splenocyte culture supernatant was analyzed to determine the levels of various cytokines, including IFN-γ, TNF-α, IL-17A, IL-4, IL-10, and IL-6 (**B**) Gating strategy for CD4^+^ T cell subtypes. (**C**) The percentages of IFN-γ+ cells, IL-4^+^ cells, and IL-17^+^ cells in the CD3^+^CD4^+^ cells were measured by flow cytometry. Data are representative as mean ± SEM, with *n* = 7–8. Student’s *t*-test, * *p* < 0.05, ** *p* < 0.01.

We investigated the immune-modulating activity of HDRsEf1 on T lymphocyte populations in the spleen. However, our analysis did not reveal any significant discrepancies in the proportions of CD3^+^ cells, CD4^+^ T cells, and CD8^+^ T cells between the HDRsEf1 and control groups (Figure S1). To gain further insight into the impact of HDRsEf1 administration on T cell functionality, we assessed the secretion profiles of cytokines in splenocytes from mice treated with either HDRsEf1 or PBS. Our findings demonstrated a noteworthy elevation in the levels of IFN-γ in the culture supernatant of splenocytes from the HDRsEf1 group compared to the control group. Nevertheless, there were no notable variations observed in the levels of IL-4, IL-10, IL-6, TNF-α, and IL-17A between the two groups (Figure 2A). These results mean that HDRsEf1 may activate Th1 cells but has no effects on Th2 and Th17 cells. Additionally, we employed flow cytometry and the gating strategy depicted in Figure 2B to examine the subtypes of T helper cells in the spleen. The proportion of Th1 cells within the CD4^+^ T cell population, as well as the Th1/Th2 ratio, demonstrated a significant increase in the HDRsEf1 group, aligning with the cytokine production characteristics observed in the splenocyte culture supernatant (Figure 2C). However, there were no notable changes observed in the Th2 and Th17 cells, which is consistent with the results of cytokines. These findings suggest that the utilization of HDRsEf1 can effectively promote Th1 differentiation and function in the spleen.

### 3.3. HDRsEf1 Strongly Promotes IFN-γ Production in Splenocytes In Vitro

**Figure 3:**
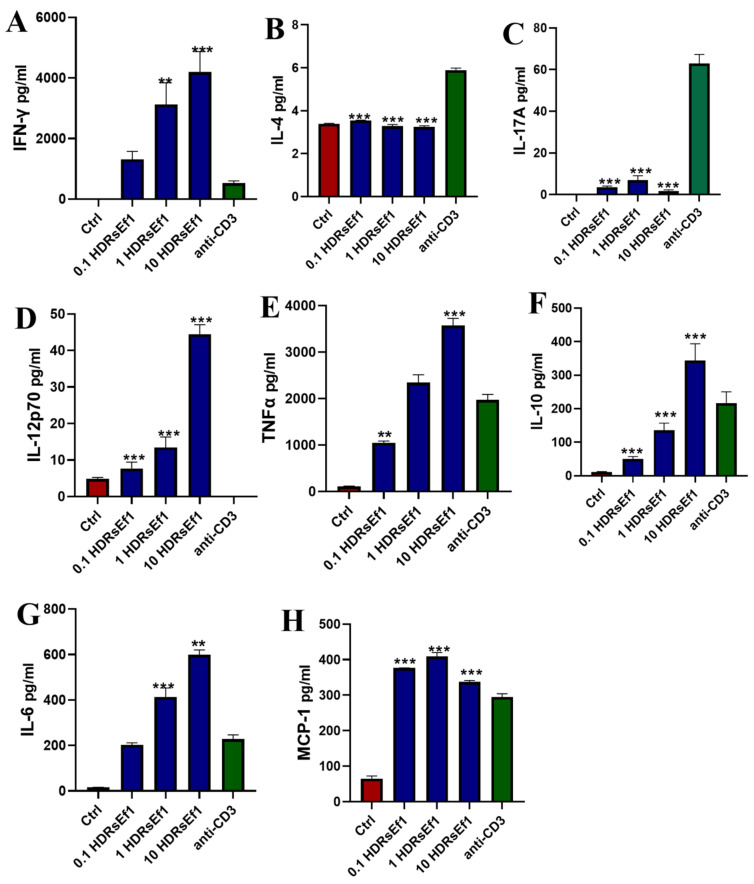
HDRsEf1 promotes splenocytes to produce IFN-γ in vitro. Splenocytes were cultured with or without the presence of anti-CD3 mAb and HDRsEf1 for 72 h. (**A**–**H**) Expression levels of IFN-γ, IL-12p70, TNF-α, IL-17A, IL-4, IL-10, IL-6, and MCP-1 in the culture supernatant measured. Data represent the mean ± SEM in three independent experiments. The *p*-values were calculated with one-way ANOVA for multiple comparisons. 0.1 HDRsEf1, 1 HDRsEf1, and 10 HDRsEf1 represent ratios of 0.1:1, 1:1, and 10:1, respectively, for HDRsEf1 to splenocyte. ** *p* < 0.01, *** *p* < 0.001 vs. anti-CD3 group.

In order to assess the immunomodulatory role of HDRsEf1, we carried out an in vitro investigation to evaluate the production of cytokines in splenocytes when stimulated using heat-inactivated HDRsEf1. According to our research, we discovered that the production of IFN-γ, IL-12p70, IL-10, TNF-α, and IL-6 significantly increased in a dose-dependent manner when exposed to heat-inactivated HDRsEf1. Similar levels of IL-10, TNF-α, IL-6, and MCP-1 synthesis were observed upon stimulation with HDRsEf1 and anti-CD3 mAb (Figure 3E–H). However, HDRsEf1 demonstrated significantly increased production of Th1 cytokines IFN-γ and IL-12p70 compared to anti-CD3 mAb (Figure 3A,D). Notably, the concentration of IFN-γ induced by HDRsEf1 at a medium dose was nearly 10 times higher than that induced by anti-CD3 mAb. Furthermore, the induction of IL-4 and IL-17A by HDRsEf1 was minimal (Figure 3B,C). These results indicate that the PAMPs of HDRsEf1 have the potential to activate the spleen’s Th1 immune response.

### 3.4. HDRsEf1 Induces IFN-γ in a Strain-Specific Manner

**Figure 4:**
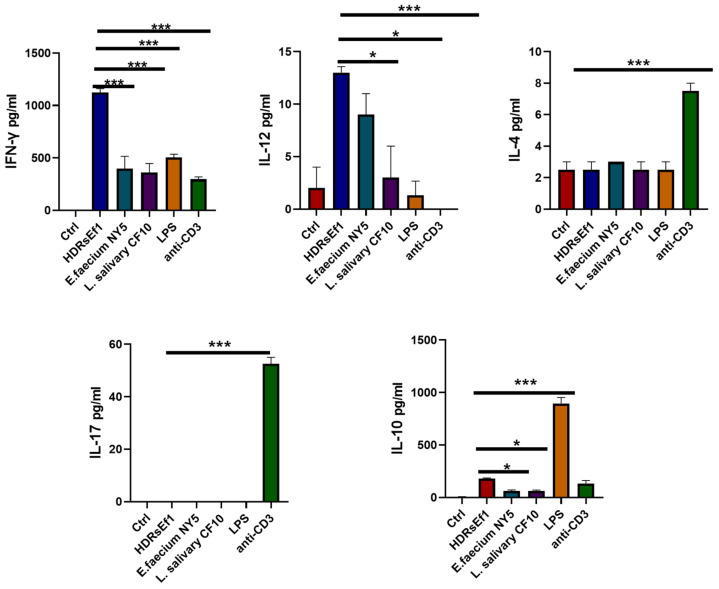
HDRsEf1 induces IFN-γ in a strain-specific manner. Splenocytes from SPF mice were cultured with or without the presence of HDRsEf1, E. faecium NY5, L. salivarius CF10, LPS, and anti-CD3Amb for 3 days. All the strains were cocultured with splenocytes at a ratio of 1:1. Cytokines secreted in the supernatant were measured. The *p*-values were calculated with one-way ANOVA for multiple comparisons, * *p* < 0.05, *** *p* < 0.001.

Various studies have demonstrated that the impact of microorganisms on the immune system is dependent on the specific strain [31,32]. Additionally, certain species of lactic acid bacteria, such as *Lactobacillus salivarius*, have been found to promote the production of IFN-γ in vitro [33]. In this study, we compared the ability of HDRsEf1, E. faecium NY5, *Lactobacillus salivarius* CF10, and LPS to induce cytokines in splenocytes. Our research revealed that all strains and LPS had the capability to induce the production of IFN-γ to varying degrees. However, HDRsEf1 was observed to induce significantly higher levels of IFN-γ compared to the other strains and LPS (Figure 4). Additionally, the production of IL-12p70 induced by HDRsEf1 was also significantly higher than that induced by L. salivarius CF10, LPS, and anti-CD3mAb (Figure 4). In contrast, all these strains and LPS failed to induce the production of IL-4 and IL-17A in splenocytes (Figure 4). LPS induced significant production of IL-10, with the concentration being notably higher compared to other strains and anti-CD3 mAb (Figure 4).

### 3.5. HDRsEf1 Promotes IL-12p70 in BMDCs and Spleen Tissue

**Figure 5:**
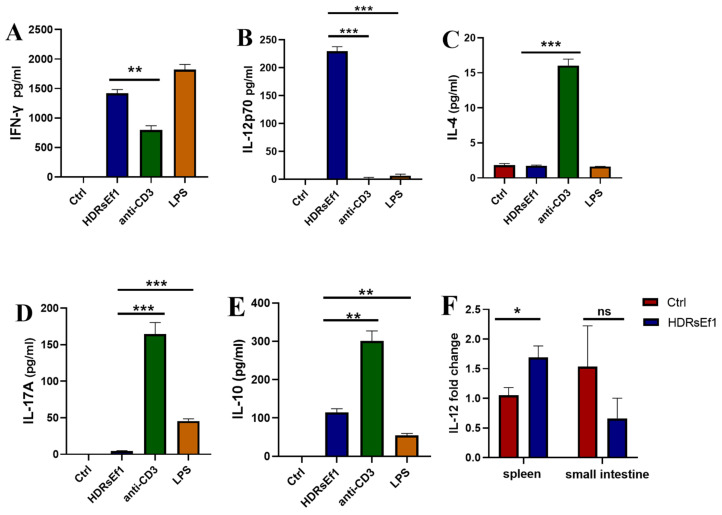
HDRsEf1 promotes IL-12 in BMDCs and in spleen tissue. (**A**–**E**) BMDCs and CD4^+^ T cells were cocultured and stimulated with LPS, anti-CD3 mAb, or HDRsEf1 for 3 days, and cytokines in the supernatant were measured. (**F**) IL-12 gene expression in the spleen and intestinal tissue from mice treated with HDRsEf1 or PBS was examined (*n* = 5–7). The *p*-values were calculated with one-way ANOVA for multiple comparisons or Student’s *t*-test for two groups, * *p* < 0.05, ** *p* < 0.01, *** *p* < 0.001.

In order to corroborate the activation of CD4^+^ T cells, we evaluated the secretion of cytokines in the supernatants by analyzing the cocultures of BMDCs and CD4^+^ T cells in the presence or absence of HDRsEf1, LPS, and anti-CD3 mAb. Our results showed that cocultures of BMDCs and CD4^+^ T cells with HDRsEf1 induced significantly higher concentrations of IFN-γ production compared to anti-CD3 mAb, and comparable levels to those induced by LPS (Figure 5A). Additionally, in comparison to LPS and anti-CD3 mAb, HDRsEf1 significantly increased the production of IL-12p70 (Figure 5B). However, the production of IL-4 and IL-17A induced by HDRsEf1 was significantly lower than that induced by LPS and anti-CD3 mA (Figure 5C,D). Furthermore, the upregulation of the IL-12 gene was confirmed in spleen tissue by RT-PCR in mice treated with HDRsEf1 but not in intestinal tissue. These results suggest that HDRsEf1 can induce the generation of IL-12, which in turn promotes the generation of Th1 cells (Figure 5F).

### 3.6. HDRsEf1 Enhances Resistance to Salmonella Typhimurium Infection in Mice

**Figure 6:**
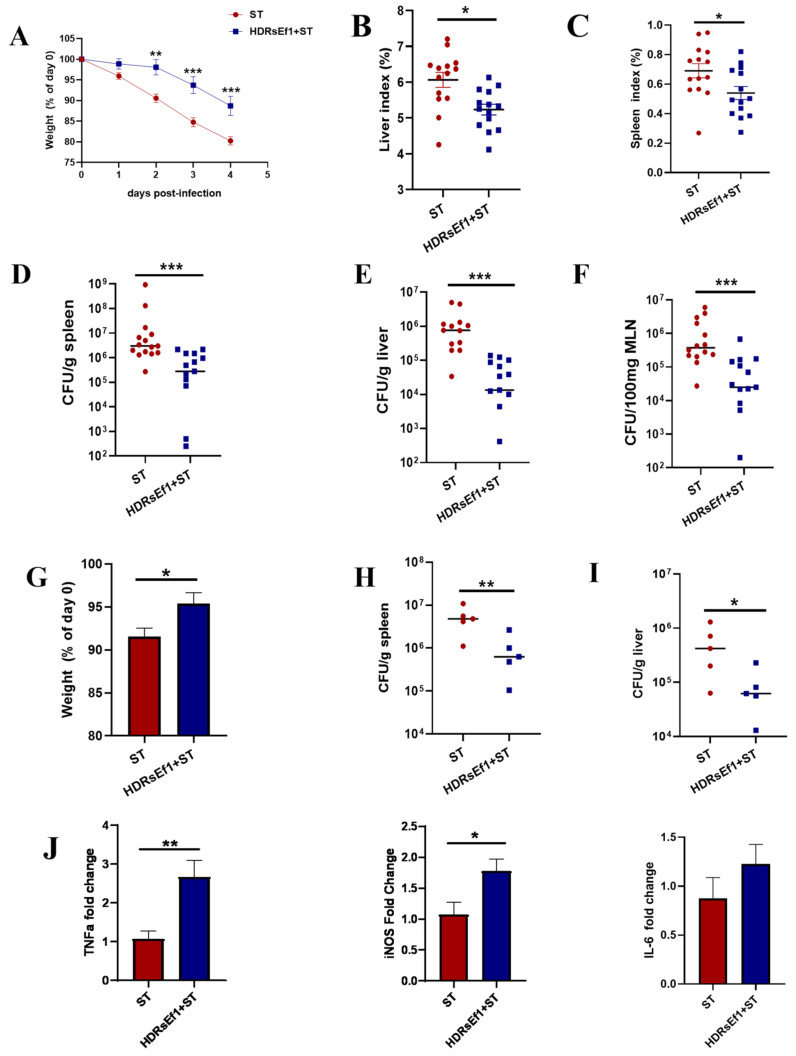
HDRsEf1 alleviating *S*. *Typhimurium* infection in mice. (**A**–**F**) Weight loss, organ index, and organ tissue bacterial burden of mice in each group challenged with *S*. *Typhimurium* orally, *n* = 15 per group. (**G**–**I**) Weight loss and organ tissue bacterial burden of mice in each group challenged with *S*. *Typhimurium* via intravenous injection, *n* = 5 per group. (**J**) Expression levels of the genes TNFα, iNOS, and IL-6 in spleen tissue 12 h post infection, *n* = 4–5 per group. Data represent the mean ± SEM (**A**–**C**,**G**,**J**) or median (**D**–**F**,**H**,**I**) from three independent experiments. The *p*-values were calculated with Student’s *t*-test (**A**–**C**,**G**) or the Mann–Whitney test (**D**–**F**,**H**,**I**). ST, *S. Typhimurium*. * *p* < 0.05, ** *p* < 0.01, *** *p* < 0.001.

Next, we investigated the potential of HDRsEf1 in mitigating infections caused by *S*. *Typhimurium*, an intracellular pathogen known to cause systemic infection. The results showed that mice pretreated with HDRsEf1 were less vulnerable to *S*. *Typhimurium* oral infection. This was evidenced by the lower body weight loss in the HDRsEf1 group compared to the PBS group (Figure 6A). Additionally, gross examination of organs from infected mice indicated that HDRsEf1 pretreatment led to reduced splenomegaly and hepatomegaly (Figure 6B,C). *S*. *Typhimurium* colonization was quantified in extraintestinal organs, such as the liver, spleen, and mesenteric lymph nodes (MLN). The uninfected animals were found to be free of *S*. *Typhimurium* in their extraintestinal organs. The pretreatment with HDRsEf1 in infected mice resulted in a notable decrease in *S*. *Typhimurium* quantities in the liver, spleen, and MLN when compared to the group treated with PBS (Figure 6D–F). To exclude the direct inhibitory effect of HDRsEf1 on *S*. *Typhimurium* in the intestine, mice were infected with *S*. *Typhimurium* via intravenous injection. Interestingly, body weight loss and *S*. *Typhimurium* load in the spleen and liver were significantly reduced in the HDRsEf1 group compared to the PBS group (Figure 6G–I). Next, we detected the expression of inflammatory factors and iNOS in spleen tissue during the early stage of infection (12 h post infection). We observed a significant increase in the expression levels of the genes iNOS and TNFα in mice treated with HDRsEf1 compared to the control group (Figure 6J). These findings suggest that HDRsEf1 can enhance antibacterial defenses by modulating immune responses.

## 4. Discussion

Research has shown that intestinal microorganisms play a crucial role in influencing the immune system, which encompasses the development and maturation of immune organs, along with the differentiation of immune cells [1,2]. It is widely acknowledged that germ-free mice and antibiotic-treated mice exhibit notable histological and immunological differences in the spleen, thymus, and intestinal lymph tissue compared to conventional mice [34]. Certain microorganisms can profoundly impact CD4^+^ T cells, leading to changes in the host’s susceptibility to various diseases [3,4,35]. There has been a notable increase in interest regarding the utilization of probiotics as a strategy for regulating immune function and averting illness [15,36,37]. Consequently, comprehending the potential consequences and mechanisms of probiotics on the immune system holds paramount significance in order to thoroughly investigate the advantages they offer. In this investigation, we have established that the colonization capacity of HDRsEf1 within the intestine is highly efficient, leading to the enhanced development of spleen morphology and stimulation of Th1 cells within the spleen. Additionally, our findings suggest that HDRsEf1 enhances resistance to systemic infection caused by *S*. *Typhimurium*. These results highlight the significant contribution of HDRsEf1 in the development of the immune system.

Commensal-derived MAMPs play a crucial role in regulating the immune system. MAMPs have been found to not only interact with immune cells in the intestines, but they also can enter the host’s circulatory system and affect the function of immune cells in other organs outside of the intestines [9,35]. Pattern recognition receptors on DCs in immune organs recognize MAMPs, leading to the induction of distinct Th cell subtypes [30,38]. Our results indicate that HDRsEf1 is capable of inducing Th1 cells and promoting the production of IFN-γ in both in vivo and in vitro experiments. This suggests that the MAMPs from HDRsEf1 can effectively activate antigen-presenting cells and promote the development of Th1 cells. It is well known that the differentiation of naïve CD4^+^ T cells to Th1 cells is facilitated by IL-12, primarily produced by DCs. Previous findings have reported that *Bacteroides fragilis* could induce Th1 production in an IL-12-dependent manner [39,40]. Similarly, in this investigation, it was also observed that HDRsEf1 prompted a notable augmentation in the generation of IL-12 within DCs. This finding suggests that HDRsEf1 facilitates the differentiation of Th1 cells within the spleen via the involvement of IL-12. Our study revealed upregulation of the IL-12 gene in spleen tissue, while intestinal tissue did not show any upregulation. This disparity could be attributed to the different immune environments in these tissues. The spleen was found to be more prone to producing IL-12 and IFN-γ, while the intestines exhibited a greater degree of immunological tolerance [41]. Previous studies have demonstrated that certain components of Gram-positive probiotics, such as peptidoglycan, are sufficient to regulate immune responses [42,43]. Therefore, additional research is required to confirm the specific components produced by HDRsEf1 that impact systemic Th1 immunity.

*S*. *Typhimurium* is a zoonotic pathogen that is capable of infecting multiple animal species, including humans, pigs, and poultry [44]. Apart from causing inflammation in the intestines, *S*. *Typhimurium* can also invade extraintestinal organs like the spleen and survive in macrophages and dendritic cells [29,45]. Studies have shown that Th1 cells and IFN-γ can improve macrophages’ ability to eliminate pathogens, particularly intracellular infections, such as *S*. *Typhimurium* and *Mycobacterium tuberculosis* [5,46,47]. Moreover, studies have demonstrated that the gut microbiome can stimulate the generation of Th1 lymphocytes and IFN-γ, which can confer defense against *S. Typhimurium* infection [5,15]. Our study demonstrated that HDRsEf1 effectively alleviates pathological symptoms and decreases the bacterial load in the spleen and liver caused by *S. Typhimurium* systemic infection. These results indicate that HDRsEf1 can enhance its antibacterial defenses through immune regulation. Indeed, during the initial phase of the infection, it was observed that the expression of iNOS and TNFα genes in the spleen was significantly enhanced by HDRsEf1. iNOS and TNFα are highly expressed in macrophages when they are challenged with pathogens, as they possess strong antimicrobial activity [48]. The rapid production of iNOS and TNFα is crucial for the host to effectively control the infection. Previous research has demonstrated that endogenous IL-12 and IFN-γ prime macrophages to produce iNOS and TNFα more promptly in response to pathogen invasion [27,28]. Therefore, our data strongly suggest that HDRsEf1 may prime macrophages for antibacterial defenses by the induction of IL-12 and Th1 cells.

Taken together, this study demonstrates that MAMPs from HDRsEf1 regulate the expression of IL-12 and induce a systemic Th1 immune response, thereby enhancing antibacterial defenses against systemic *S. Typhimurium* infection. These findings suggest that HDRsEf1 is an excellent candidate for the development of immunobiotic functional foods or feed additives to prevent systemic infections caused by pathogens, especially in individuals with compromised immunity. In addition, the in vivo evaluation of the ability of inactivated HDRsEf1 to promote immune defense is also an interesting point for further research.

## Data Availability

The data presented in this study are available on request from the corresponding author.

## Supplementary Materials

The following supporting information can be downloaded at: https://www.mdpi.com/article/10.3390/nu15194241/s1. Figure S1: Effects of HDRsEf1 on spleen T lymphocyte populations. The proportions of CD3^+^ cells, CD4^+^ T cells, and CD8^+^ T cells in CD45^+^ cells in the spleen of the control and HDRsEf1-treated animals were determined using flow cytometry. Data are representative of two independent experiments, with *n* = 7–8 as mean ± SEM. Student’s *t*-test, ns, nonsignificant. Table S1: The primer sequences for RT-qPCR.
