# Supplementary material for: Enterococcus faecium HDRsEf1 Promotes Systemic Th1 Responses and Enhances Resistance to Salmonella Typhimurium Infection
Source: Nutrients. 2023 Sep 30;15(19):4241. doi: 10.3390/nu15194241 (PMC10574401; doi:10.3390/nu15194241)
Supplement: Supplementary file 1 [file nutrients-15-04241-s001.zip › nutrients-2640607-supplementary.pdf]

Table S1. The primer sequences for RT-qPCR.

Genes	Primer sequences (5′-3′)
β-actin	F: GGCTGTATTCCCCTCCATCG<br>R: CCAGTTGGTAACAATGCCATGT
TNFα	F: CCCTCCAGAAAAGACACCATG<br>R: GCCACAAGCAGGAATGAGAAG
iNOS	F: CTGCAGCACTTGGATCAGGAACCTG<br>R: GGGAGTAGCCTGTGTGCACCTGGAA
IL-6	F: GTCGGAGGCTTAATTACACA<br>R: TTTTCTGCAAGTGCATCATC
IL-12	F:ACCTGCTGAAGACCACAGATGACA<br>R:TAGCCAGGCAACTCTCGTTCTTGT

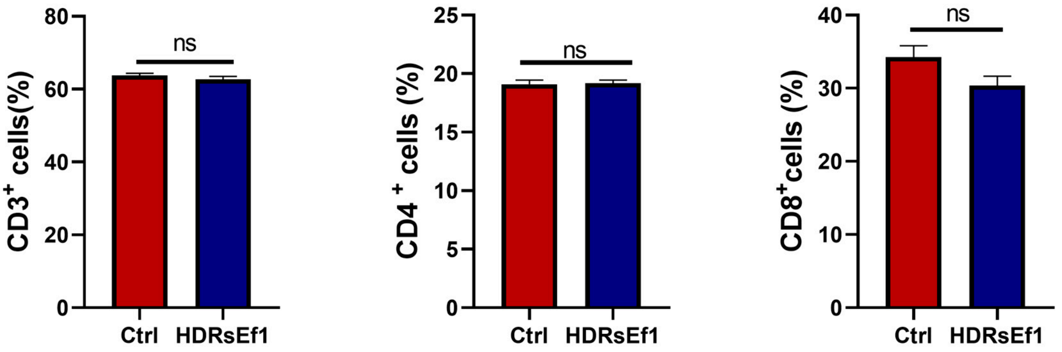

Figure S1. Effects of HDRsEf1 on spleen T lymphocyte populations. Proportion of $CD3^+$ cells, $CD4^+$T cells and $CD8^+$T cells in $CD45^+$ cells in the spleen of control and HDRsEf1-treated animals were determined by flow cytometry. Data are representative of two independent experiments with n = 7–8 as mean ± SEM. Student's t-test, ns, non-significant.
